# Recent Advances in Nanostructured Perovskite Oxide Synthesis and Application for Electrocatalysis

**DOI:** 10.3390/nano15060472

**Published:** 2025-03-20

**Authors:** Xiaofeng Xue, Bowen Li

**Affiliations:** Institute for Frontier Science, Nanjing University of Aeronautics and Astronautics, Nanjing 210016, China

**Keywords:** perovskite oxides, nanostructure engineering, water splitting, CO_2_ conversion

## Abstract

Nanostructured materials have garnered significant attention for their unique properties, such as the high surface area and enhanced reactivity, making them ideal for electrocatalysis. Among these, perovskite oxides, with compositional and structural flexibility, stand out for their remarkable catalytic performance in energy conversion and storage technologies. Their diverse composition and tunable electronic structures make them promising candidates for key electrochemical reactions, including the oxygen evolution reaction (OER), hydrogen evolution reaction (HER), and carbon dioxide reduction (CO_2_RR). Nanostructured perovskites offer advantages such as high intrinsic activity and enhanced mass/charge transport, which are crucial for improving electrocatalytic performance. In view of the rapid development of nanostructured perovskites over past few decades, this review aims to provide a detailed evaluation of their synthesis methods, including the templating (soft, hard, colloidal), hydrothermal treatments, electrospinning, and deposition approaches. In addition, in-depth evaluations of the fundamentals, synthetic strategies, and applications of nanostructured perovskite oxides for OER, HER, and CO_2_RR are highlighted. While progress has been made, further research is needed to expand the synthetic methods to create more complex perovskite structures and improve the mass-specific activity and stability. This review offers insights into the potential of nanostructured perovskite oxides in electrocatalysis and provides potential perspectives for the ongoing research endeavor on the nanostructural engineering of perovskites.

## 1. Introduction

Nanostructured materials have gained tremendous attention in recent years due to their unique and often superior properties compared to their bulk counterparts [1]. Their high surface area to volume ratio, enhanced catalytic activity, and tunable electronic, optical, and mechanical properties make them ideal candidates for a wide range of applications, particularly in the fields of catalysis, energy storage, and environmental applications [2,3,4]. In catalysis applications, nanostructuring materials can significantly improve the reactivity of catalysts by providing more active sites for reactions and facilitating faster electron and ion transport. Additionally, the size and shape of nanomaterials can be finely controlled, allowing for the design of materials with specific properties tailored to particular applications [5,6,7].

Perovskite oxides, as a class of nanostructured materials, have gained considerable attention in recent years due to their remarkable structural flexibility and excellent catalytic performance [8,9]. The perovskite structure, characterized by the general formula ABO_3_, consists of a lattice made up of a larger cation (A), a smaller cation (B), and an oxygen anion (O) in a cubic or distorted octahedral arrangement. This structure offers significant versatility, as both the A and B sites can be substituted with a wide range of metal ions, allowing for the tuning of the material’s electronic, catalytic, and thermal properties. Perovskite oxides can be synthesized in various forms, such as thin films, nanoparticles, and nanowires, providing further opportunities for tailoring their properties for specific catalytic applications [10,11]. In recent years, perovskite oxides have emerged as highly efficient catalysts in a variety of electrochemical reactions, including the oxygen evolution reaction (OER), hydrogen evolution reaction (HER), and carbon dioxide reduction (CO_2_RR) [12,13,14]. Their ability to facilitate these critical reactions makes them a promising candidate for the development of clean energy technologies, as they can contribute to both energy generation and storage, as well as to the mitigation of environmental issues like CO_2_ emissions.

**Figure 1:**
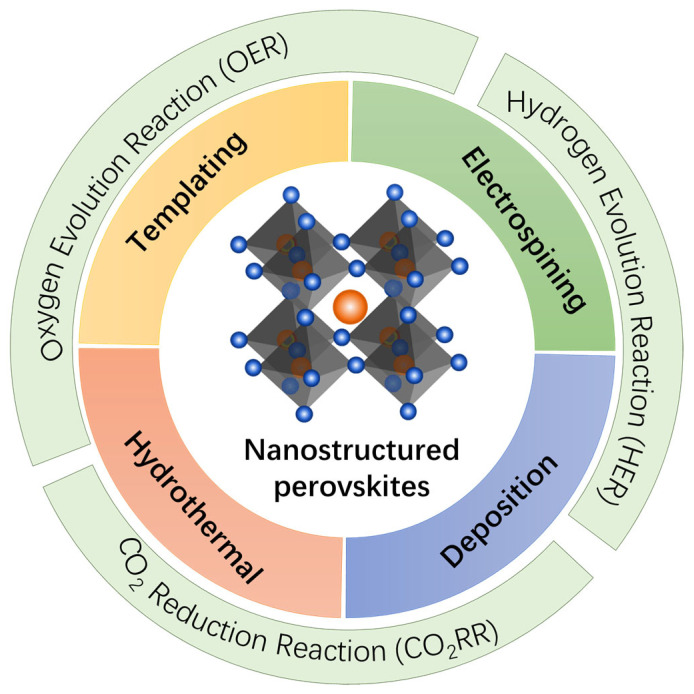
Schematic illustration of the synthetic strategies for nanostructured perovskites and their applications in the OER, HER and CO_2_RR.

Herein, this review focuses on the recent advances in the synthesis of nanostructured perovskite oxides and their application in the oxygen evaluation reaction (OER), hydrogen evolution reaction (HER), and CO_2_ reduction reaction (CO_2_RR) (Figure 1). We provide an in-depth discussion of the various synthetic strategies employed to prepare these nanostructured perovskites, including templating methods (such as soft, hard, and colloidal templating), hydrothermal treatments, electrospinning, and deposition techniques. This review also delves into the performance of nanostructured perovskite oxides in these electrocatalytic reactions, highlighting the performance enhancement associated with the nanostructural engineering. Finally, we discuss the current challenges and future directions for research in this field, emphasizing the need for new synthesis methods that can produce complex and multi-component perovskites, as well as the exploration of additional electrocatalytic reactions, such as nitrogen reduction (N_2_RR) and nitrate reduction (NO_3_RR), where perovskites hold significant promise. Through this comprehensive review, we aim to provide insights into the potential of nanostructured perovskite oxides as next-generation catalysts for sustainable energy conversion and environmental applications.

## 2. Fundamentals of Perovskite Oxides for Electrocatalysis

Perovskite oxides (ABO_3_), with their unique crystal structure and tunable composition, have gained significant attention as electrocatalysts in recent years [8,15]. Their versatile properties make them suitable candidates for a wide range of energy-related applications. The appeal of perovskite oxides lies in their highly customizable nature, enabling the optimization of catalytic performance through careful tuning of the A-site and B-site elements and their electronic structure. This section will provide a detailed overview of perovskite oxide catalysts, including their crystal structure, the role of compositional tuning, and the advantages and challenges of their application in electrocatalysis.

The perovskite structure is characterized by a distinct three-dimensional arrangement of atoms, where the general formula is ABO_3_ [16]. In this structure, the A-site cation (typically a larger ion) is coordinated by twelve oxygen ions in a cubic arrangement, forming a larger unit cell, while the B-site cation (typically smaller in size) is coordinated by six oxygen ions, forming an octahedral geometry. This framework is crucial to the structural flexibility and adaptability of perovskite materials. The ideal perovskite structure, typically referred to as the “ideal cubic perovskite”, can be distorted depending on the size and charge of the A- and B-site ions, as well as external factors like the temperature and pressure [17]. This distortion can lead to a variety of substructures, including tetragonal, orthorhombic, or rhombohedral phases, each influencing the material’s electronic, magnetic, and catalytic properties [18]. The oxygen sublattice within the perovskite oxide also plays a vital role in determining its catalytic behavior, as oxygen vacancy formation and migration are critical to many electrocatalytic reactions [19].

One of the key advantages of perovskite oxide catalysts is their compositional tunability. Both the A-site and B-site cations can be modified to enhance the catalytic activity, stability, and selectivity for specific electrocatalytic reactions [20,21]. The flexibility to substitute different cations at both sites allows the design of catalysts with tailored properties. The A-site cation in perovskites is often a rare earth metal or alkaline earth metal, such as La, Sr, Ba, or Ca. These cations are primarily responsible for the lattice size, ionic conductivity, and charge compensation in the material. By substituting different elements at the A-site, the electronic structure of the perovskite can be modulated, affecting properties such as the oxidation state, electronic conductivity, and ionic conductivity. The B-site cation typically comprises transition metals. The B-site metal controls the catalytic activity of the perovskite, particularly in reactions involving oxygen species. Transition metal cations such as Ni^2^⁺, Co^3^⁺, or Fe^3^⁺ can have varying oxidation states, making them highly versatile for catalysis.

## 3. Synthetic Strategies for Nanostructured Perovskites

The conventional synthesis of perovskite oxides can be achieved through methods like solid-state calcination and sol–gel processing, both of which are commonly used but have inherent limitations, particularly in terms of the surface area and catalytic performance [21,22]. In the solid-state calcination method, metal oxides or carbonates are mixed in stoichiometric ratios and heated at high temperatures. This high-temperature treatment facilitates the formation of the perovskite structure, driving off volatile components and enabling the crystalline phase to form. The sol–gel method, on the other hand, involves preparing a homogeneous solution (sol) of metal salts or precursors, followed by gelation to form a solid network, which is then dried and calcined to obtain the final perovskite oxide. This approach provides better control over the material’s stoichiometry and homogeneity compared to solid-state calcination. However, high calcination temperatures often cause particle agglomeration, reducing the effective surface area and, consequently, the material’s catalytic efficiency. In view of this, numerous design strategies have been developed to synthesize perovskite oxides at a nanoscale [23,24] in order to increase the specific surface area and catalytic efficiency. The characterization of nanostructured perovskites typically involves transmission electron microscopy (TEM) and scanning electron microscopy (SEM) to analyze the morphology, particle size, and structural features at the nanoscale. Brunauer–Emmett–Teller (BET) surface area analysis is commonly used to determine the surface area, porosity, and textural properties, which are critical for applications in catalysis, energy storage, and optoelectronics. These techniques provide essential insights into the material’s structural integrity, surface characteristics, and overall performance, aiding in the optimization of the synthesis methods for specific applications. In this section, the typical approaches, including templating, hydrothermal treatment, electrospinning, and deposition approaches, will be discussed in detail.

### 3.1. Synthesis of Nanostructured Perovskites by the Templating Method

Soft templating is primarily based on the self-assembly of surfactants in the presence of inorganic precursors to form mesostructured perovskite oxides. The most commonly used technique within this approach is the evaporation-induced self-assembly (EISA) method [24], which involves several key stages. The first step involves preparing a homogeneous sol containing both surfactants and metal precursors. Surfactants such as cationic (e.g., cetyltrimethylammonium chloride, CTAC), anionic (e.g., C_16_H_33_SO_3_H), or non-ionic surfactants (e.g., Pluronic F123) are typically used to direct the self-assembly process. The inorganic metal precursors are usually metal salts like nitrates or acetates dissolved in a solvent such as ethanol or water. The sol solution is then exposed to a controlled evaporation process, usually by dip-coating or spin-coating, where the solvents are evaporated under mild heating conditions. As the solvent evaporates, surfactant molecules aggregate into micelles, creating the mesostructured framework. During this process, the metal ions are also incorporated into the self-assembled micellar structures. As the solvent evaporates further, the inorganic precursors begin to condense and form a network within the micellar structure. The surfactants stabilize the inorganic material and guide the formation of mesoporous frameworks. After the evaporation step, the organic surfactant template is removed by calcination at moderate temperatures (typically 300–500 °C) to leave behind the mesoporous perovskite oxide. This calcination process also leads to the crystallization of the oxide phase.

**Figure 2:**
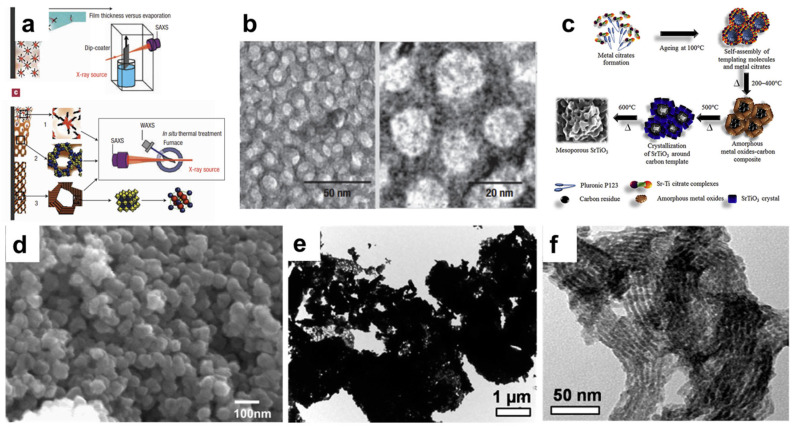
(**a**) Schematic illustration of the synthesis of SrTiO_3_ by soft-templating and dip-coating. (**b**) Representative TEM images of mesoporous SrTiO_3_ [25]. (**c**) Schematic illustration demonstrating the role of citric acid in assisting the formation of mesoporous SrTiO_3_ [29]. (**d**) Representative SEM image of BaTiO_3_ nanoparticles synthesized by the precipitation method [30]. (**e**) Representative TEM image of bulk LaNiO_3_. (**f**) Representative TEM image of mesoporous LaNiO_3_ synthesized using an SBA-15 hard template [38].

In 2004, Grosso et al. demonstrated the formation of mesoporous SrTiO_3_ perovskite using a commercial organic template and dip-coating evaporation approach (Figure 2a,b) [25]. Subsequently, other perovskites, like NaTaO_3_ and BiFeO_3_, with nanoporsosity have also been developed using a similar approach [26,27]. In addition to surfactants, chelating agents like citric acid could be introduced into the sol to assist in the formation of nanostructures [27,28]. Lertpanyapornchai et al. introduced citric acid as a complexing agent during the synthesis of SrTiO_3_ via the EISA approach, and they discovered that citric acid could promote the synthesis of highly crystalline SrTiO_3_ nanoparticles (Figure 2c) [29]. With the presence of both citric acid and Pluronic P123 (as a non-ionic surfactant), the synthesized mesoporous SrTiO_3_ has demonstrated the highest structural integrity and BET surface area (41.5 m^2^/g), which is much higher than its bulk SrTiO_3_ counterparts (<5 m^2^/g). In some cases where the perovskite precursor has strong hydrolysis abilities, precipitation, instead of evaporation, could be used to synthesize nanostructured perovskites [30,31]. Hou et al. demonstrated such a solution-based soft-templating method in preparing BaTiO_3_ nanoparticles (Figure 2d). Using cetyltrimethylammonium chloride (C16TMAC) as the cationic surfactant, nanosized BaTiO_3_ powder has been synthesized and the specific surface area was measured to be ~50 m^2^/g [30].

For mesoporous structures to remain intact, the calcination temperatures need to be controlled. Perovskite oxides, especially perovskites with structural and compositional complexity, often require a high temperature for crystallization, which can lead to the collapse of the mesostructure or formation of secondary phases. Moreover, the heterogeneous solubility of metal precursors during solvent evaporation could lead to phase separation, especially in multi-metal perovskite systems, leading to the non-homogeneous distribution of metal ions and the formation of secondary phases. As such, the synthesis of nanostructured perovskites by soft templating is not widely reported in the literature and is mainly limited to Ti-based materials.

Hard templating, on the other hand, is based on the use of rigid templates (usually mesoporous silica or mesoporous carbon) to create well-ordered nanoporous structures [32]. This method typically involves the infiltration of metal precursors into the template’s pores, followed by calcination to form the oxide phase [33]. The template is then removed using chemical etching (acidic or basic) to leave behind the mesoporous structure. For a start, a mesoporous material, such as SBA-15, KIT-6, or mesoporous carbon (e.g., CMK-1), is selected as the template. These materials have well-defined pore structures and high surface areas, which are ideal for templating purposes. Subsequently, a precursor solution is prepared by dissolving metal salts (e.g., nitrates or acetates of La, Fe, Co, Ni, etc.) in a solvent such as water or ethanol. In some cases, chelating agents like citric acid are added to enhance the uniform dispersion of the metal ions. The template is then soaked in this precursor solution to ensure that the pores are fully impregnated. The solution may be applied using methods like impregnation under vacuum or capillary action. After impregnation, the template is dried to remove excess solvent. The drying step may occur under mild conditions to prevent any unwanted phase separation or crystallization of the metal precursor before calcination. The metal-impregnated template is heated in a furnace at high temperatures in an air, inert or oxygen-rich atmosphere. This process induces the formation of perovskite oxide nanoparticles. Carbon templates can be simultaneously removed during calcination, while silica templates are removed by etching with NaOH or HF.

Mesoporous silica is often chosen because of its high thermal stability and ability to form ordered structures [34,35,36,37]. For instance, Nair et al. synthesized nanoporous LaNiO_3_ using SBA-15 as a hard template [38]. Compared with bulk samples, LaNiO_3_ synthesized with an SBA-15 hard template has clearly demonstrated the open channels characteristic of SBA-15 (Figure 2e,f). The specific surface area has also increased from 10 m^2^/g to 150 m^2^/g. The removal of the silica template often requires harsh acidic or basic treatments, and incomplete removal could cause pore blockage and other structural damages. As such, porous carbon is another actively explored alternative to silica materials to be used as hard templates. In particular, carbon materials could be easily removed during the calcination steps, and no acidic or basic treatment is required to remove the template. Lima et al. synthesized nanostructured LaFeO_3_ and LaFe_0.6_Co_0.4_O_3_ from porous carbon templates [39]. After impregnation with precursor solution, calcination was carried out at 800 °C, simultaneously resulting in the formation of perovskite crystals and the removal of carbon template. In addition to porous silica and carbon templates, nanosized particles could also be used as hard templates. For example, Zhang et al. synthesized honeycomb-shaped LaMnO_3_ using carbon spheres as a hard template [40]. With the removal of the carbon spheres during calcination, macroporosity was created within the LaMnO_3_ crystals and the corresponding BET surface area reached 38 m^2^/g.

Colloidal crystal templating could be seen as a special variety of the hard-templating method. It relies on using highly ordered polymer microspheres (e.g., polystyrene or PMMA) as templates to form three-dimensionally ordered macroporous (3DOM) structures [41,42]. These 3DOM materials offer significant advantages in terms of the mass transport and surface area. First, monodisperse colloidal polymer spheres (e.g., PS or PMMA) are prepared, typically through emulsion polymerization, and then arranged into a highly ordered close-packed structure, often through a simple sedimentation, evaporation or centrifugation process. The ordered template is then infiltrated with a precursor solution, usually consisting of metal nitrates dissolved in solvents such as ethanol, methanol, or ethylene glycol. The precursor solution is carefully introduced into the interstitial spaces between the polymer spheres. This can be achieved through simple impregnation or with the assistance of a vacuum. After the infiltration process, the precursor solution is solidified to form a metal oxide phase. This may involve curing the precursor at low temperatures to ensure the homogeneous formation of a perovskite oxide. After solidification, the polymer template is removed by calcination at high temperatures (typically around 700 to 800 °C). The organic polymer is completely decomposed, leaving behind the 3DOM perovskite oxide. This process typically requires careful temperature control to avoid the collapse of the porous structure.

**Figure 3:**
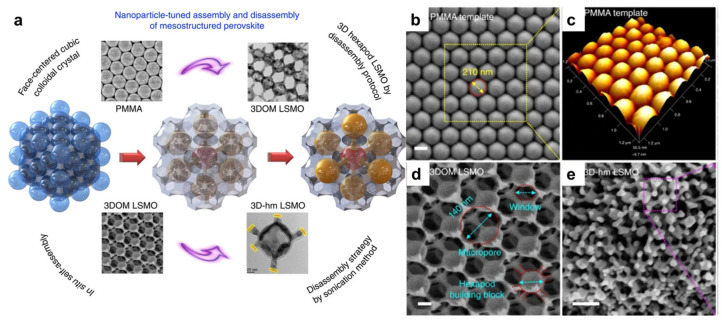
(**a**) Schematic illustration of the synthesis and controlled disassembly of the 3DOM structure. Representative SEM images of (**b**) the PMMA template, (**c**) 3DOM La_0.6_Sr_0.4_MnO_3_ and (**d**,**e**) the disassembled nanoparticles [50].

Using PMMA as the colloidal template, Chi et al. first demonstrated the formation of 3DOM La_0.7_Ca_0.3_MnO_3_ perovskite. While the PMMA spheres have a diameter of ~400 nm, the resulting macropores were measured to be ~340 nm, implying shrinkage during the calcination process [43]. 3DOM perovskites with other compositions, including LaCo*_x_*Fe_1−*x*_O_3_, La_0.7_Ca_0.3−*x*_Sr*_x_*MnO_3_, La_0.6_Sr_0.4_FeO_3−*δ*_, Eu_1–*x*_Sr*_x_*FeO_3_, have also been reported using PMMA templates [44,45,46,47]. There have also been reports of 3DOM perovskites synthesized using PS spheres as templates, though the lower thermal stability could cause the collapse of porous structures during calcination [48,49]. Moreover, it is interesting to note that these 3DOM networks could be disassembled into nanoparticles. Wang et al. reported the controlled disassembly of 3DOM La_0.6_Sr_0.4_MnO_3_ under sonication., forming hexapod nanoparticles (Figure 3) [50]. This has exposed new crystal faces to act as highly active sites for the methane combustion reaction.

Each of the three templating methods—soft template, hard template, and colloidal crystal—offers a unique pathway for synthesizing nanostructured perovskite oxides. While the soft template method is versatile and relatively simple, its challenges with phase control and scalability limit its applicability. The hard template method excels in producing well-ordered materials but faces difficulties with precursor infiltration and template removal. The colloidal crystal template method can yield materials with large, interconnected pores, but its high cost and the fragility of the templates are significant drawbacks. Ongoing improvements to precursor infiltration techniques and template removal processes, and the development of hybrid templating methods, are likely to address these challenges and expand the range of applications for these nanostructured perovskite oxides.

### 3.2. Synthesis of Nanostructured Perovskites by Hydrothermal Treatment

Hydrothermal synthesis, a widely employed technique for the fabrication of nanostructured oxides, involves the crystallization of materials from aqueous solutions at elevated temperatures and pressures [51,52]. This method has gained significant attention due to its simplicity, scalability, and ability to produce high-purity, well-defined nanostructures. It allows for the controlled manipulation of the particle size, morphology, and phase purity, which are crucial for optimizing the properties of nanostructured materials, particularly for applications in catalysis. Hydrothermal synthesis involves several steps, including nucleation, growth, and aggregation. During the process, metal ions are dissolved in the solvent and subject to complex reactions, such as hydrolysis and condensation, that lead to the formation of metal–oxygen bonds. The nanoparticles nucleate from supersaturated solutions and grow into well-defined nanostructures. For perovskites, in particular, the samples collected after hydrothermal treatment are often intermediate precursors, and subsequent calcination is required to obtain perovskite nanostructures with high crystallinity.

**Figure 4:**
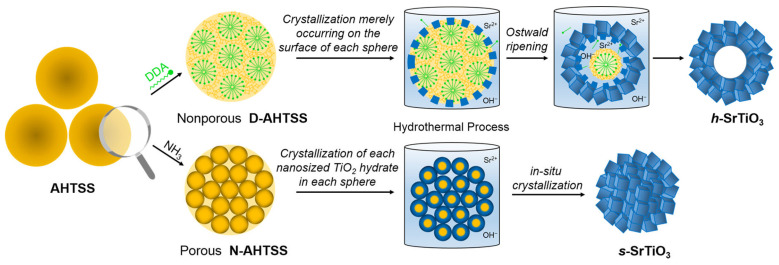
Schematic illustration of the synthesis and of hollow ATiO_3_ obtained via hydrothermal treatment [62].

Unlike the templating methods that are commonly used to synthesize porous perovskites, the morphology of the nanostructures resulting from hydrothermal treatment can be fine-tuned by various parameters, including the concentration of precursors, reaction time, temperature, and pH, leading to the formation of various shapes, such as nanorods, nanocubes, nanospheres and nanosheets [52,53,54]. For instance, Ge et al. synthesized La(Co_0.55_Mn_0.45_)_0.99_O_3−*δ*_ nanorods under hydrothermal conditions [55]. Using citric acid and uric acid as surfactants, Ogunniran et al. synthesized nanostructured Nd_0.7_Co_0.3_FeO_3_ by hydrothermal treatment followed by calcination [56]. When designing catalysis for electrocatalytic applications, hydrothermal treatments could easily integrate nanostructured perovskites with graphene and its derivatives, resulting in the formation of hybrid catalysts [57,58,59]. Apart from nanoparticles of various shapes, porous perovskites could also be synthesized from hydrothermal treatments [60,61]. Using amorphous TiO_2_ as sacrificial templates, Pan et al. developed a general synthetic strategy for producing porous/hollow ATiO_3_ perovskites (Figure 4) [62]. Hollow spheres of SrTiO_3_, BaTiO_3_, and CaTiO_3_ were believed to form via the Ostwald ripening process under hydrothermal conditions.

### 3.3. Synthesis of Nanostructured Perovskites by Electrospinning

**Figure 5:**
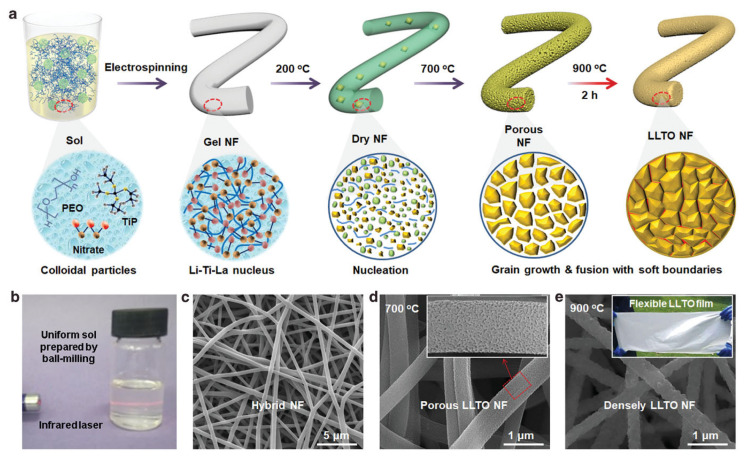
(**a**) Procedures for using electrospinning followed by calcination to fabricate Li_0.35_La_0.55_TiO_3_ nanofibers. (**b**) A clear and stable sol precursor prepared by ball-milling for 0.5 h. (**c**–**e**) SEM images of the Li_0.35_La_0.55_TiO_3_ sample at different stages [66].

Electrospinning is a versatile and efficient method for fabricating nanostructured materials, including perovskite oxides, by applying a high voltage to a precursor solution [10,13,63]. The process involves the extrusion of a polymeric solution containing the metal precursors through a needle, which creates a charged jet of the solution. As the jet travels through the air, the solvent evaporates, and the metal salts or organometallic compounds undergo precipitation, resulting in the formation of fine nanofibers. Polymers are commonly used to provide the necessary viscosity for the jetting process and ensure that the nanofibers retain their integrity during collection. These fibers can then be calcined to remove any organic components and convert the metal salts into the desired perovskite oxide phase. The electrospinning technique is particularly beneficial for producing perovskite oxide nanofibers with a high surface area and porosity [64,65]. Xu et al. used the electrospinning technique to synthesize porous La_0.75_Sr_0.25_MnO_3_ nanotubes. The high porosity and surface area of the synthesized La_0.75_Sr_0.25_MnO_3_ sample resulted in excellent performance in lithium–oxygen batteries [63]. Recently, Li et al. further demonstrated the control of nanofiber porosity by controlling the crystal nucleation and gradient calcination process, forming flexible Li_0.35_La0.55TiO_3_ nanofibers with controlled pore defects (Figure 5) [66]. Similar to templating methods, the addition of chelating agents (e.g., citric acid) is important for ensuring the synthesis of phase pure perovskites [67,68].

Unlike the above strategies, where only ABO_3_ perovskites could be synthesized, the electrospinning method could be used to synthesize perovskite nanofibers with a complex crystal structure. Hildebrandt et al. synthesized perovskite nanofibers with a layered crystal structure, namely Ba_5_Ta_4_O_15_, Ba_5_Ta_2_Nb_2_O_15_ and Ba_5_Nb_4_O_15_, revealing the key role of the amorphous barium carbonate intermediate in ensuring the nanofiber formation [69]. Bu et al. used electrospinning to prepare PrBa_0.5_Sr_0.5_Co_2–*x*_Fe*_x_*O_5+δ_ double perovskite with mesoporous nanofiber morphology, and they applied it as a bifunctional catalyst for a zinc–air battery [70]. More recently, Zhou et al. further optimized the operation conditions to synthesize PrBa_0.8_Ca_0.2_Co_2_O_5+δ_ double perovskite with 3D-structured porosity and used it for zinc–air battery applications [71].

### 3.4. Synthesis of Nanostructured Perovskites by Deposition Approaches

Physical vapor deposition (PVD) is a widely used technique for the deposition of thin films and nanostructured materials, including perovskite oxides [72,73,74,75]. PVD involves the vaporization of a solid or liquid precursor material in a vacuum chamber, followed by the condensation of the vapor onto a substrate to form a thin film or nanostructured material. The PVD method includes various sub-techniques, such as sputtering, evaporation, and laser ablation, which differ in the manner by which the material is vaporized and deposited. The key advantage of PVD lies in its ability to produce high-quality, uniform films and nanostructures, often with minimal defects, which is crucial for the performance of devices such as fuel cells, superconductors, and electronic devices. PVD can be particularly useful in the fabrication of perovskite oxide thin films and nanoparticles with precise control over the thickness, crystallinity, and morphology. Using laser ablation as the evaporation method, pulsed laser deposition (PLD) has been adopted for deposition of La_0.8_Sr_0.2_CoO_3_ and (La_0.5_Sr_0.5_)_2_CoO_4_ superlattices with precisely controlled thickness onto (STO) (001) substrates [76]. This system was used as a model platform for understanding the interfacial electronic structures and explaining the superior oxygen reduction reaction reactivities. Chen et al. employed magnetron sputtering to deposit an amorphous Ba_0.5_Sr_0.5_Co_0.8_Fe_0.2_O_3−δ_ perovskite layer onto a surface-oxidized nickel substrate. Compared with bulk crystallites, this physically deposited amorphous layer has shown a two order of magnitude enhancement of the reactivity for the OER [77].

Electrodeposition is an electrochemical method used for the synthesis of nanostructured perovskites, offering advantages in terms of the low cost, simplicity, and scalability [78,79]. In electrodeposition, a metal or oxide film is deposited onto a conductive substrate by applying a potential to drive the reduction or oxidation of metal ions in solution, forming a solid film. A variety of conducting substrates have been used for electrodeposition, including glassy carbon, carbon papers, indium–tin oxide (ITO), metals, etc. For the deposition of perovskites, in particular, Pt is the most commonly used substrate in the literature, resulting in the formation of LaCrO_3_, LaCoO_3_ and LaMnO_3_ thin films [80,81,82]. As a replacement for the expensive Pt substrate, a recent study synthesized LaCrO_3_ on a stainless-steel substrate by calcination of the electrodeposited mixed-metal hydroxide coating layer [83]. In addition, Zhang et al. coated LaCo_0.8_Fe_0.2_O_3_ perovskite nanoparticles on an nickel foam substrate by electrodeposition under an O_2_-saturated electrolyte [84].

### 3.5. Evaluation of Various Synthetic Approaches

The synthesis of nanostructured perovskites varies in scalability, cost-effectiveness, and energy efficiency across different methods. Templating techniques, particularly soft and hard templating, face scalability challenges due to the complex template removal, while colloidal templating offers better scalability with moderate energy consumption. Hydrothermal treatments provide moderate scalability but are energy-intensive due to the high temperatures and pressures. Electrospinning has limited throughput and high energy demands but enables precise nanostructure formation. PVD offers high-quality thin films with excellent control over the composition and crystallinity but is limited by the scalability and high energy costs. In contrast, electrodeposition is more scalable, cost-effective, and energy-efficient, making it suitable for large-scale and flexible perovskite applications. However, it may have limitations in terms of the film uniformity and composition control compared to PVD. The synthesis of nanostructured perovskites remains largely at the laboratory scale, with specific methods required to achieve the desired properties for particular applications. However, these approaches often lack the flexibility needed for large-scale production. To bridge the gap between research and industrial application, it is crucial to develop more generalized and scalable synthesis strategies that ensure a high yield, reproducibility, and cost-effectiveness while maintaining the material performance and structural integrity. Additionally, direct comparisons of different synthesis methods regarding the yield, purity, cost, and energy consumption are typically not reported in the literature, making it difficult to assess their industrial feasibility. A more systematic evaluation of these factors is needed to guide the development of scalable and efficient manufacturing techniques.

## 4. Application of Nanostructured Perovskite Oxides in Electrocatalytic Reactions

Electrocatalytic reactions play a crucial role in addressing some of the most pressing challenges in terms of energy conversion and environmental sustainability. Among these, the OER, HER and CO_2_RR are particularly significant due to their central roles in renewable energy production, energy storage, and greenhouse gas mitigation [85,86]. The OER and HER are key reactions in water splitting, a process essential for generating green hydrogen as a clean energy carrier, while the CO_2_RR offers a potential solution for reducing atmospheric CO_2_ levels by converting it into valuable chemicals or fuels. The development of efficient, cost-effective, and durable electrocatalysts for these reactions is critical for advancing clean energy technologies, fostering the transition to a low-carbon economy, and mitigating the impacts of climate change. Owning to the drastic advancements of halide perovskite in solar cell applications, the application of halide perovskites in electrocatalytic applications have also been investigated [87]. However, the structural instability, especially when subjected to aqueous electrolytes, has greatly limited their potential applications. Perovskite oxides, on the other hand, with much improved robustness, have been extensively explored for electrocatalytic applications. In this section, novel nanostructured perovskite catalysts designed for the OER, HER and CO_2_RR are evaluated, respectively.

### 4.1. Nanostructured Perovskite Oxides for the Oxygen Evolution Reaction (OER)

The OER plays a central role in water splitting and other electrocatalytic applications, which is essential for sustainable hydrogen production [88,89]. The OER involves the oxidation of water molecules to produce oxygen gas, protons, and electrons. The key factors influencing the OER include the reaction conditions (e.g., pH, temperature, and applied voltage), the electronic structure of the catalysts, and the nature of the active sites. Typically, the OER is governed by the four-electron process, but the pathway is influenced by factors like the surface structure and the electronic state of the catalyst. The mechanism of the OER has been widely debated, with two primary models proposed to explain the reaction pathway: the adsorption evolution mechanism (AEM) and the lattice oxygen mechanism (LOM) [90,91]. In the AEM, the reaction proceeds through a series of steps where water molecules are adsorbed onto the catalyst surface and oxygen species (O*) are formed through deprotonation and oxidation. These adsorbed oxygen species undergo successive oxidation steps, leading to the formation of an oxygen–oxygen bond (O–O), which results in the release of O_2_. In contrast, the LOM suggests that the oxygen evolution is facilitated by the participation of lattice oxygen atoms from the catalyst itself. In this mechanism, lattice oxygen atoms are oxidized, forming O_2_ as they are released from the catalyst surface. The LOM is particularly relevant for transition metal oxides, where the metal’s lattice oxygen plays a pivotal role in the OER process [92]. Both mechanisms highlight the importance of active sites, electron transfer, and proton coupling in achieving efficient oxygen evolution, but the exact pathway often depends on the catalyst’s composition, structure, and operating conditions. The catalyst reactivity for the oxygen evolution reaction (OER) is determined by key performance metrics, including the overpotential, which indicates the additional voltage required to drive the reaction; Tafel slope, which reflects the reaction kinetics and charge transfer efficiency; current density, which represents the catalytic activity per unit electrode area; turnover frequency (TOF), which measures the intrinsic activity per active site; and mass activity, which assesses the efficiency of the catalyst relative to its mass. Meanwhile, the stability of the catalyst is typically evaluated through repeated cyclic voltammetry (CV) scans, which monitor changes in electrochemical behavior over multiple cycles, or chronoamperometry and chronopotentiometry tests, which assess long-term operational stability by measuring the current or potential over extended periods under constant voltage or current conditions. These evaluations are crucial for determining the practical applicability and durability of OER catalysts in real-world electrochemical systems.

RuO_2_ and IrO_2_ are often considered the benchmark catalysts for the OER due to their excellent catalytic performance [93,94]. However, their high cost and limited availability hinder their widespread use in large-scale applications. As a result, there has been significant interest in exploring alternative catalysts that are both more affordable and scalable. Perovskite-based materials, composed of transition metal oxides, have emerged as promising replacements due to their lower cost, versatility, and high intrinsic catalytic activity for the OER [12]. Perovskites, particularly those incorporating elements like cobalt, nickel, and iron, exhibit high activity due to their favorable electronic structure and the ability to tune their properties through composition and synthesis methods. Despite their promising catalytic properties, perovskites suffer from relatively low mass-specific activity due to the low surface area resulting from conventional solid-state and sol–gel synthesis.

**Figure 6:**
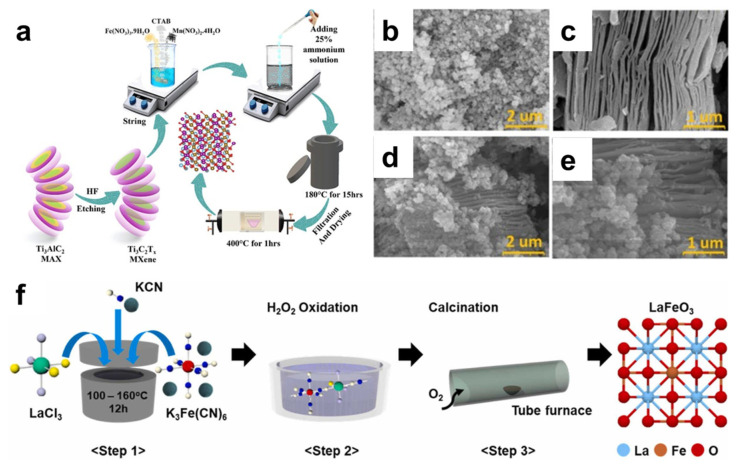
(**a**) Proposed preparation route for MXene@MnFeO_3_ hybrid nanocomposites. Representative FESEM images of (**b**) MnFeO_3_, (**c**) MXene, and (**d**,**e**) the MXene@MnFeO_3_ hybrid nanostructure [96]. (**f**) Schematic illustration of the synthesis steps for LaFeO_3_ via the cyanogel–peroxo-complex [97].

To overcome this challenge, the development of nanostructured perovskite catalysts has gained significant attention. By synthesizing perovskite nanoparticles via the hydrothermal method, the produced nanoparticles with a reduced particle size could effectively increase the specific surface area and catalytic reactivity of perovskites [95]. For instance, Kim et al. synthesized porous LaCoO_3_ hollow spheres by the hydrothermal route and used them for alkaline OER application. Compared with bulk counterparts, hollow LaCoO_3_ has shown a six times increase in the current density, owing to its higher specific surface area and highly active amorphous surface species [54]. Sheikh et al. also used the hydrothermal method to produce MnFeO_3_ nanoparticles. In their study, MXene powders were introduced to the autoclave during the hydrothermal process, forming MXene@MnFeO_3_ hybrid nanostructures (Figure 6a–e) [96]. This hybrid catalyst has shown promising OER activity, measuring an overpotential of 235 mV at 10 mA/cm^2^. Kim et al. presented a hydrothermal-based step-wise strategy for the synthesis of LaFeO_3_ nanoparticles in a low temperature range (300–500 °C). The hydrothermal treatment, followed by H_2_O_2_ oxidation, leads to the formation of key metastable intermediate, namely the cyanogel–peroxo-complex, that allows the formation of highly crystalline LaFeO_3_ nanoparticles with ideal ABO_3_ stoichiometry. When evaluated for the alkaline OER, the catalyst has shown an overpotential of 438 mV at 100 mA/cm^2^, surpassing that of the benchmark IrO_x_/C commercial catalyst (Figure 6f) [97].

**Figure 7:**
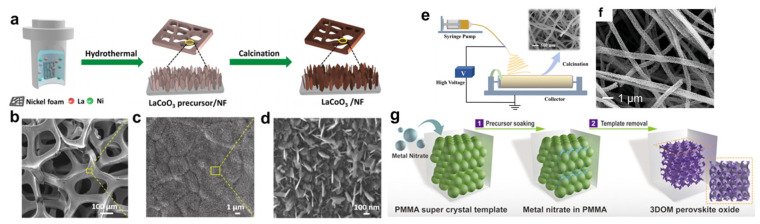
(**a**) Schematic diagram of the synthesis process for LaCoO_3_/NF. (**b**–**d**) SEM images of LaCoO_3_/NF at different magnifications [102]. (**e**) Schematic diagram of the synthesis process for La_0.5_Sr_0.5_Fe_1−x_NixO_3−*δ*_ nanofiber by electrospinning [107]. (**f**) Representative SEM image of La_0.5_Sr_0.5_Fe_1−x_NixO_3−*δ*_ nanofiber. (**g**) Schematic illustration of the synthesis of 3DOM LaFeO_3_ for OER application [109].

Apart from synthesizing nanoparticles with a reduced size, the formation of thin films with nanosized thickness could further enhance the exposure of catalytically active sites [98,99,100]. Yang et al. used a stepwise hydrothermal–calcination method to synthesize 2D SrIrO_3_ nanosheets with a ~5 nm thickness [101]. First, hydrothermal treatment was used to obtain a precursor polymer containing Sr and Ir species, while the subsequent calcination in air resulted in the formation of SrIrO_3_ with nanosheet morphology. This catalyst has exhibited stable OER reactivity in an acidic electrolyte, with no significant decay over 5000 repeated CV cycles. By introducing nickel foam into the precursor solution, Zhao et al. managed to grow 2D LaCoO_3_ nanoarrays on nickel foam (Figure 7a–d). This composite catalyst was evaluated for the alkaline OER, and the overpotential at 10 mA/cm^2^ is 342 mV, significantly lower than that of bulk LaCoO_3_ (390 mV) [102]. Apart from hydrothermal synthesis, physical vapor deposition and electrodeposition have also been used for synthesizing perovskite nanosheets in order to enhance their OER reactivity [84,103]. For example, magnetron sputtering has been used to synthesize Ba_0.5_Sr_0.5_Co_0.8_Fe_0.2_O_3−δ_ perovskite nanosheets. By adjusting the sputtering time, thin films of ~1 nm thickness could be fabricated, demonstrating much higher OER reactivity than the bulk counterparts [77].

Porous perovskites with nanosized shells could be seen as a special variety of nanosheet morphology, and the application of these meso/macroporous perovskites for the effective OER has also been reported in the literature. Porous perovskite nanofibers synthesized by electrospinning have been widely explored as an effective OER catalyst [104,105,106]. Li et al. adopted the electrospinning approach to synthesize La_0.5_Sr_0.5_Fe_1−x_Ni_x_O_3−*δ*_ nanofibers (Figure 7e,f) [107]. Compared with the bulk sample, this nanostructured perovskite has a higher specific surface area and faster mass transportation. This catalyst not only demonstrates outstanding reactivity in a typical three-electrode system (e.g., achieving 295 mV overpotential at 10 mA/cm^2^) but has also been used as an anodic material for an anion-exchange membrane water electrolyzer (AEMWE), outperforming the commercial benchmark RuO_2_ anode. Synthesis of 3DOM perovskites by the colloidal templating method could also effectively increase the surface area and improve the mass transport efficiency [108]. As a demonstration, Dai et al. used PMMA templates to synthesize 3DOM LaFeO_3_ (Figure 7g), achieving two-fold enhancement of the OER reactivity from the bulk sample [109]. The authors also claimed that the high surface area and good charge/mass transport properties are the key factors responsible for the performance enhancement.

### 4.2. Nanostructured Perovskite Oxides for the Hydrogen Evolution Reaction (HER)

Being the cathodic half-reaction in water splitting, HER is the direct process used to produce green H_2_. The HER involves the reduction of protons (H⁺) to form hydrogen gas (H_2_), a clean and energy-dense fuel. The HER typically follows a two-electron transfer process, but its mechanism is heavily influenced by the catalyst’s surface structure and the interaction between the protons and the catalyst [110,111]. The mechanism of the HER has been extensively studied, with two main models proposed: the Volmer–Heyrovsky mechanism and the Volmer–Tafel mechanism [112]. In the Volmer–Heyrovsky mechanism, the HER proceeds in two steps. The first step involves the adsorption of a proton (H⁺) onto the catalyst surface. This step requires the proton to be reduced by gaining an electron from the cathode. The second step involves the combination of an adsorbed hydrogen atom (H*) with another proton (H⁺) and an electron (e⁻) from the electrode, leading to the formation of hydrogen gas (H_2_). This step is called the Heyrovsky reaction. For the Volmer–Tafel mechanism, the same Volmer step takes place to produce adsorbed H*. The second step in the Volmer–Tafel mechanism, however, involves the direct combination of two adsorbed hydrogen atoms (H*) to form hydrogen gas (H_2_). The efficiency of the HER is determined by factors such as the catalyst’s hydrogen binding energy, electronic conductivity, and surface morphology. Catalyst reactivity and stability evaluations typically follow the same set of criteria for OER evaluation.

While Pt/C is the commercial benchmark catalyst for the HER, research has focused on finding potential replacements from non-noble metals for similar reasons stated for the OER application [113]. Unlike the OER, however, perovskites are typically less active for HER applications, and the application of nanostructured perovskites is less reported in the literature [114,115,116]. Nonetheless, Li et al. reported a combined electrospinning and phosphatizing strategy to synthesize nanostructured perovskites with high HER reactivity [117]. To be specific, Pr_0.5_La_0.5_BaCo_2_O_5+*δ*_ nanofiber was first synthesized by electrospinning with DMF/PVP solution. Subsequently, the sample was calcined with NaH_2_PO_2_ under N_2_ flow to introduce a P dopant into the perovskite structure. This P-doped Pr_0.5_La_0.5_BaCo_2_O_5+*δ*_ has been tested as highly active for the HER, comparable to the benchmark Pt/C commercial catalyst. The high HER reactivity was believed to be the combination of the desired 1D structure, the synergistic charge transfer between Co and P, and the ideal H* adsorption energy.

### 4.3. Nanostructured Perovskite Oxides for the CO_2_ Reduction Reaction (CO_2_RR)

CO_2_ utilization is a critical process in the context of sustainable energy systems and climate change mitigation. CO_2_ catalytic conversion, by the thermal-, photocatalytic-, and electrocatalytic- routes, provides a sustainable solution for mitigating carbon emissions by transforming CO_2_ into valuable chemicals and fuels, contributing to carbon-neutral energy systems and addressing climate change [118,119,120]. In particular, the CO_2_RR involves the electrochemical conversion of carbon dioxide (CO_2_), a major greenhouse gas, into valuable chemicals and fuels. This process offers a dual advantage: it reduces atmospheric CO_2_ levels while simultaneously producing renewable energy carriers, contributing to a circular carbon economy. By transforming CO_2_ into a variety of useful chemicals, the CO_2_RR plays an important role in closing the carbon loop, enabling the recycling of CO_2_ back into the economy rather than allowing it to accumulate in the atmosphere, which is a key contributor to global warming. One of the most intriguing aspects of the CO_2_RR is its wide range of potential products, depending on the specific reaction conditions and catalysts used, including CO, formic acid, methanol, ethylene, ethanol, etc. [86,121]. The ability to selectively produce different C_1_, C_2_, and even C_3_ products from CO_2_ makes the CO_2_RR a highly flexible and powerful process for creating sustainable, low-carbon chemicals and fuels. However, the challenge lies in tuning the catalyst and reaction conditions to achieve the desired product with high selectivity and efficiency [120]. For example, the formation of C_2_ and C_3_ products generally requires the coupling of C_1_ intermediates (CO or formate), a step that involves complex electron and proton transfer processes [122,123]. Therefore, the design of catalysts that can facilitate these multi-step reactions while minimizing the side reactions is essential for improving CO_2_RR performance. When evaluating the performance of CO_2_RR catalysts, in addition to the previously discussed standards for the OER and HER, product selectivity is of particular concern. The Faradaic efficiency (FE) for the CO_2_RR is a key metric that quantifies how efficiently the electrical charge supplied to the system is utilized for converting CO_2_ into the desired products. It is defined as the ratio of the charge used to produce a specific product to the total charge passed through the system. A high FE for a specific product indicates selective and efficient conversion of CO_2_, which is crucial for optimizing the catalyst performance and energy utilization in electrochemical CO_2_ reduction applications.

**Figure 8:**
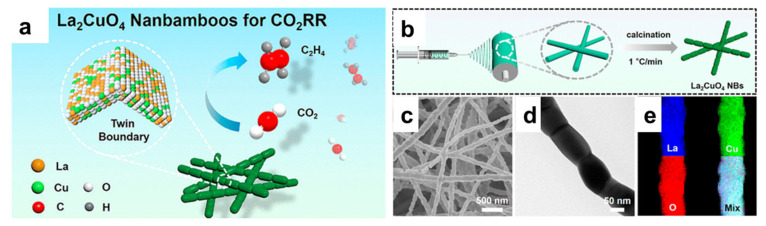
(**a**) Schematic illustration of the application of La_2_CuO_4_ nanobamboos for the CO_2_RR. (**b**) Schematic of the synthesis of La_2_CuO_4_ nanobamboos by electrospinning. (**c**–**e**) Representative SEM, TEM, and EDX mappings of La_2_CuO_4_ nanobamboos [132].

The development of catalysts for the CO_2_RR is at a critical stage, with significant progress made in understanding the fundamental mechanisms and improving the performance of catalysts. Currently, a wide range of materials have been explored for CO_2_RR catalysts, including metal-based catalysts, metal oxide catalysts, carbon-based catalysts, and hybrid materials. Among these, transition metals (e.g., Cu, Ag, Sn, Bi, etc.) and their alloys have garnered the most attention due to their ability to convert CO_2_ into valuable C_1_ and C_2_ products [124,125,126,127]. Perovskite oxides have been reported as efficient CO2RR catalysts in producing CO, formate, and other C_2+_ products, while attempts have been made to further improve their catalytic performance by nanostructural engineering [128,129,130,131]. Utilizing the electrospinning technique, Wang et al. synthesized La_2_CuO_4_ Ruddlesden–Popper perovskite with a 1D nanobamboo structure (Figure 8) [132]. When tested for the CO_2_RR, a high product selectivity toward C_2_H_4_ was achieved, with Faradaic efficiency of 60%. This is drastically different from its bulk counterpart, which favors the production of CO (faradaic efficiency < 90%). In situ spectrometry and theoretical calculations propose that the lattice strain associated with the nanobamboo structure and the preferential exposure of the (113) surfaces are the two key factors responsible for the difference in product selectivity.

## 5. Conclusions and Outlook

In conclusion, this review summarizes the development of various nanostructured perovskite synthetic strategies, such as templating methods, hydrothermal treatments, electrospinning, and deposition techniques. The application of these nanostructured perovskites to emerging electrocatalysis applications, namely the OER, HER and CO_2_RR, has been examined, focusing on how the nanostructural engineering enables these perovskite materials to act as effective catalysts with enhanced reactivity and stability. However, there are still several challenges that need to be addressed in order to fully exploit the potential of nanostructured perovskite oxides in electrocatalysis. The synthesis methods for these materials remain limited, with most approaches primarily focused on ABO_3_ perovskites, while perovskites with more complex structures (e.g., double/triple and layered structures) remain underexplored. Developing new, more versatile and scalable synthetic techniques is essential for creating more sophisticated perovskite materials with superior catalytic properties. In addition, the exact structure–performance correlation for nanostructured perovskites in electrocatalysis is still unclear, making it challenging to predict and optimize their catalytic behavior. The complexity of perovskite nanostructures, including variations in the morphology, composition, atomic arrangement, and defects, can significantly impact their catalytic properties. As such, there is a pressing need for advanced theoretical calculations, such as density functional theory (DFT), to explore these correlations in greater depth. These models can offer valuable insights into the electronic structure, reaction pathways, and active sites, helping to identify the most efficient and stable configurations. Moreover, while significant progress has been made in water splitting (OER and HER) applications, the use of nanostructured perovskite oxides for the CO_2_RR is still in its infancy, requiring much more research to optimize their performance in this area. Looking forward, future research should focus on improving the mass-specific activity and long-term stability of nanostructured perovskite catalysts, especially under industrial-scale operating conditions. Exploring the use of nanostructured perovskites for other emerging electrocatalytic reactions, such as the nitrogen and nitrate reduction reactions, presents exciting new opportunities for perovskite-based materials in energy and environmental applications. Finally, interdisciplinary approaches combining materials science, electrochemistry, and computational modeling will be essential for accelerating the development of perovskite-based electrocatalysts for large-scale, real-world applications. While the performance of nanostructured perovskites in several key half-reactions, e.g., the OER, HER and CO_2_RR, is evaluated in this review, the application of such materials in electrocatalytic devices like fuel cells, electrolyzers (e.g., PEM for water splitting and MEA for the CO_2_RR), requires interdisciplinary cooperation toward milestone breakthroughs. The future of nanostructured perovskite oxides in electrocatalysis holds great promise, and ongoing advancements will play a pivotal role in the transition toward a more sustainable and carbon-neutral energy future.
